# Assessing Changes in COVID-19 Vaccine Uptake and Intentions among the Brigada Digital Latino Social Media Audience: A Repeated Cross-Sectional Study

**DOI:** 10.21203/rs.3.rs-3611896/v1

**Published:** 2023-11-20

**Authors:** Courtney Riggle-van Schagen, Elizabeth Louise Andrade, Shikha Chandarana, Nathan Lu, Anna González, Carla Favetto, Valeria Gomez, César Palacios, Manuel Díaz-Ramírez, Mark Cameron Edberg

**Affiliations:** George Washington University; George Washington University; George Washington University; George Washington University; George Washington University; George Washington University; George Washington University; Proyecto Salud; La Clínica del Pueblo; George Washington University

**Keywords:** COVID-19, vaccination, Latinos, social media campaign, community-based outreach, health information access, misinformation, language minority populations

## Abstract

**Background.:**

U.S. Latinos experienced disproportionate COVID-19 impacts in terms of morbidity and mortality. Vaccination against COVID-19 is an important strategy for mitigating health impacts, and yet, vaccine uptake was slower among U.S. Latino adults compared to other racial/ethnic groups. Vaccine hesitancy has been a significant barrier within Latino communities, and exposure to misinformation has been associated with negative attitudes toward vaccination. While some COVID-19 mitigation efforts have included community-based outreach, few studies have explored the impact of community-based digital messaging in Spanish to counter COVID-19 misinformation, build trust, and promote vaccination.

**Methods.:**

To address this gap, we conducted a one-year repeated cross-sectional study to assess changes in COVID-19 vaccine uptake, intentions, and perceived norms, as well as barriers to accessing reliable information and levels of trust in COVID-19 information sources among Latino adults exposed to *Brigada Digital de Salud* social media content. This culturally-tailored content disseminated on Facebook, Instagram, and X platforms was amplified by community health workers and partners, and focused on COVID-19 risk and prevention, vaccine safety and efficacy, and correcting related misinformation.

**Results.:**

Statistically significant increases in COVID-19 vaccination, intentions to vaccinate children, and vaccination subjective norms were observed from May 2022 (wave 1) to April 2023 (wave 2). Among perceived difficulties accessing information, respondents indicated the most difficulty in judging the reliability of COVID-19 information in the media; however, a statistically significant decrease in perceived difficulty was observed between waves. With regard to trust in COVID-19 information sources, levels of trust were highest for healthcare providers in both waves. From wave 1 to wave 2, there were statistically significant increases in trust in the FDA to ensure COVID-19 vaccine safety and trust in the federal government to ensure child COVID-19 vaccine safety.

**Conclusions.:**

Social media messaging by trusted community-based sources shows promise as a strategy for combating health misinformation and ameliorating information access gaps for language minority populations. This digital approach represents an important tool for deploying critical information to underserved populations in public health emergency and crisis contexts, and for supporting changes in attitudes, trust, and behaviors to improve health outcomes.

## Background

Throughout the COVID-19 pandemic, Latinos in the U.S. have been disproportionately impacted by SARS-CoV-2 in terms of morbidity and mortality (1–3). Within the first year of the pandemic, the Centers for Disease Control and Prevention (CDC) estimated that Latinos were 1.5 times more likely to be infected, 2.3 times more likely to be hospitalized, and 1.8 times more likely to die from COVID-19 in comparison to White, Non-Latino persons (4). These disparities have been explained, in part, by differences in comorbidity prevalence and social determinants, such as healthcare access, employment in “essential” frontline industries, and socioeconomic factors (5–9).

Vaccination is an important step in reducing the spread and severity of COVID-19. However, vaccine uptake was slower among U.S. Latino adults compared to other racial/ethnic groups (10–13), and Latino children continue to have disproportionately lower vaccination rates in some age categories. In May of 2023, 57% of Latino adults had completed a primary vaccine series while only 9.1% had received a bivalent booster dose. This was the lowest booster dose coverage across all racial/ethnic subgroups, although data on race/ethnicity was incomplete for approximately one-fifth of individuals (14). Furthermore, as of August 2023, only 28.8% of Latino children ages 5–11 years, 57.8% of those aged 12–15 years, and 70.4% of those aged 16–17 years had completed a primary vaccine series (15). U.S. Latinos have experienced disproportionate barriers to COVID-19 vaccination, such as challenges with taking time off work, limited transportation and vaccine access, language barriers surrounding vaccine information and scheduling appointments, fear of discrimination, disclosing citizenship status or deportation (14, 16–23).

Vaccine hesitancy has also been a significant barrier among Latinos (5, 10, 12, 16–18). Studies have established that exposure to COVID-19 vaccine misinformation is associated with negative attitudes towards vaccines, lower trust in science, uncertainty about source reliability, and decreased intention to vaccinate (24–27). A 2021 review of 13 studies showed that approximately one-third of Latino participants experienced vaccine hesitancy, and that hesitancy was associated with higher levels of exposure to misinformation and medical mistrust (28).

National strategies to address mistrust and increase COVID-19 vaccination rates among Latinos have emphasized the importance of community outreach and engagement; these strategies have focused on mitigating COVID-19 risk and increasing vaccine confidence among vulnerable populations (16, 29–32). Most commonly, COVID-19 educational efforts targeting U.S. Latinos have been led and implemented by local organizations. These efforts have employed community-based approaches involving community health workers, community leaders, and social media outreach (10, 33–36). Initiatives such as CDC’s WhatsApp-based *Mi Chat Sobre Vacunas COVID* and the Unidos U.S.-led *Esperanza Hope for All* campaign reached audiences on a national level (10, 37, 38).

While there have been initiatives to address COVID misinformation in U.S Latino communities, there have been a lack of studies exploring the impact of community-based digital messaging strategies that counter COVID-19 misinformation in Spanish and promote vaccine uptake. The *Brigada Digital de Salud* (Digital Health Brigade) was established in May of 2021 to address the proliferation of COVID-19 misinformation and narratives fueling vaccine hesitancy within Spanish language social media networks. Spanning across Facebook, Instagram, Twitter (now X), and TikTok, *Brigada Digital de Salud* has disseminated accessible, evidence-based, and culturally appropriate COVID-19 information in Spanish on a weekly basis (39). This study examines changes in COVID-19 vaccination and related outcomes among *Brigada Digital* Latino audience members.

## Methods

### Design.

We conducted a one-year repeated cross-sectional study to assess changes in self-reported adult and child COVID-19 vaccine uptake, intentions, and perceived norms, difficulty accessing COVID-19 information, and trust in COVID-19 information sources and government institutions to ensure vaccine safety among *Brigada Digital* Latino individuals from the Washington, DC Metropolitan (DMV) area who have been exposed to *Brigada Digital de Salud* social media content.

### Intervention.

**Figure 1:**
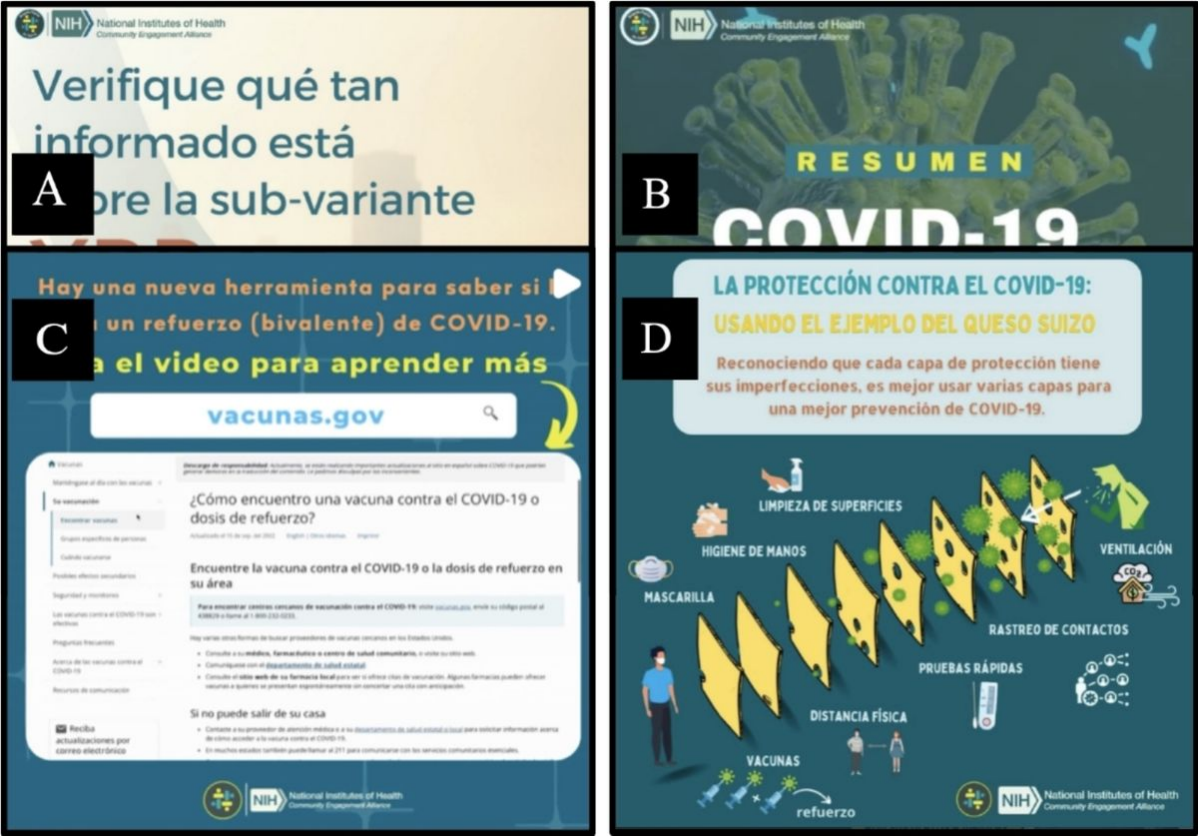
Examples of *Brigada Digital de Salud* social media content. Panel **A** is a carousel that shares information about the COVID-19 XBB.1.5 variant. Panel **B** is an informational video about public health achievements of COVID-19 vaccination. Panel **C** is a video tutorial that teaches viewers how to determine when they need a COVID-19 booster dose. Panel **D** is an infographic that visually illustrates the benefits of layered COVID-19 mitigation strategies.

Beginning in May of 2021, we developed and disseminated approximately two to three weekly social media posts in Spanish to educate audience members about: COVID-19 variants, risk, and prevention; testing; vaccine recommendations, safety, and efficacy; COVID-19 treatment options; and to promote resources for vaccination, testing, and prevention. Given the rapidly changing information landscape, *Brigada Digital* social media content also sought to provide regular news and scientific updates, explain changes in COVID-19 policies and vaccine eligibility, and correct COVID-19 misinformation. *Brigada Digital* content was developed for audiences with diverse levels of literacy and education levels, and included explanations of scientific concepts, visual illustrations, and audio narration of text. Content was delivered in varied formats, including carousels, videos, and tutorials (See Fig. 1).

From May 8, 2022 to April 5, 2023, we disseminated a total of 141 unique posts across each of the *Brigada Digital* Facebook, Instagram, X, and TikTok pages, which were then shared by a trained cadre of 10 community health workers (CHW) with their social media networks and Spanish language, Latino-oriented public DMV-based Facebook groups. *Brigada Digital* CHWs also conducted digital outreach and health promotion activities to engage audience members, answer questions, and connect community members with resources. A comprehensive discussion of *Brigada Digital* content development, topics, communication and community-based outreach strategies, and overall audience reach and engagement has been published elsewhere (39).

### Instrument and Measures.

The Common Survey 2.0 was developed by the National Institutes of Health’s Community Engagement Alliance Against COVID-19 Disparities (NIH CEAL), a national consortium of regional research collaboratives. The survey instrument included measures for sociodemographics, media consumption, adult and child COVID-19 vaccine uptake and intentions, adult booster uptake, difficulty accessing COVID-19 information, and levels of trust in COVID-19 information sources and government entities.

Sociodemographic variables included age, place of birth/origin, sex, education level, employment status, and household income. The survey instrument asked participants’ birth year, and a variable for respondent age was created by subtracting the year of survey administration (e.g. 2022) from the respondent’s reported year of birth (e.g. 2021 – 1975, yielding an age for the respondent of 46). Age categories were then created, including: 18–25, 26–35, 36–45, 46–55, 56–63, and 64+. The survey instrument included eight response options for household income, which was simplified by collapsing household income into four categories, including $15,000-$34,999, $35,000-$74,999, $75,000-$100,000>, and “declined to answer.” Likewise, the original variable for educational attainment was collapsed from eight categories to three, including “Less than high school/Some high school,” “High school graduate/GED/some college,” and “Associates, Bachelor’s or Postgraduate degree.”

Participants’ English language competency was assessed on a 5-point Likert scale from “Speaks English very well” to “Does not speak English at all,” with higher mean scores representing greater English competency. Participants were also asked whether they had ever been diagnosed with a chronic health condition and whether they had health insurance coverage. In addition, the survey included questions about time spent consuming media, social media platforms used, and sources from which they obtained COVID-19 information (i.e., healthcare provider, faith leader, news outlet, social media, federal government). Daily amount of time spent consuming media was captured using a 5-point Likert scale from “None” to “Six hours or more.” Participants were asked to indicate which social media platforms they used, for example, Facebook, WhatsApp, Twitter (now X), Instagram, TikTok, and Snapchat. Sources from where respondents obtained COVID-19 information (i.e., healthcare provider, local television channel, social media, friends or family in the U.S., state or local government, and federal government agencies) were assessed using a 4-point Likert scale with options ranging from “None of my information” to “All of my information” from each specific source. Higher means (ranging from 1–4) indicate more COVID-19 information was obtained from that particular source.

### Primary Outcomes.

Self-reported adult COVID-19 vaccine uptake, adult booster dose uptake and intentions, and COVID-19 vaccine uptake and intentions for their children under age 18 were assessed as primary outcomes. To assess adult COVID-19 vaccine uptake, participants were asked whether they had received at least one dose of the COVID-19 vaccine, with the response options including, “I received one dose of a two-dose series,” “I received both doses of a two-dose series,” “I received a one-dose vaccine,” and “I have not been vaccinated against COVID-19.” For participants indicating that they had received a one-dose vaccine or both doses of a two-dose series, they were also asked whether they had ever received a booster dose, with response options including, “I received a booster dose,” “I received more than one booster dose,” “I have not received a booster dose, but I plan to,” and “I have not received a booster dose, and I do not plan to.” Results related to vaccine intentions are reported separately from vaccine uptake results.

Among participants indicating that they were a parent/guardian of at least one child under age 18, child vaccine uptake and parent intentions to vaccinate their child(ren) was assessed by asking whether they were in favor of vaccinating their child/ren against COVID-19, with response options including, “Yes, child/ren is/are already vaccinated,” “Yes, I plan to vaccinate my child/ren,” “No, I do not plan on vaccinating my child/ren,” and “I’m unsure/undecided.” Participants were also asked about reasons for deciding to not vaccinate their child(ren) against COVID-19.

### Secondary Outcomes.

COVID-19 vaccination subjective norms, difficulty accessing COVID-19 information, trust in different sources of COVID-19 information, trust in the FDA to ensure COVID-19 vaccine safety, and trust in the federal government to ensure COVID-19 vaccine safety for children were assessed as secondary outcomes. COVID-19 vaccination subjective norms were assessed using a 4-item scale that asked participants how many people close to them thought they should get the COVID-19 vaccine, and how many of their friends, family, and community members had received the vaccine. A 4-point Likert scale ranging from “None” to “All” was used, with higher mean scores indicating a greater number of individuals.

Perceived levels of difficulty “finding needed COVID-19 information,” “finding COVID-19 information in my preferred language,” and “judging whether COVID-19 information in the media was reliable” were assessed using three survey questions. Items were assessed using a 4-point Likert scale, and response options included “Difficult,” “Somewhat difficult,” “Somewhat easy,” and “Easy,” with higher mean scores indicating greater difficulty.

Participants’ levels of trust in various sources of COVID-19 information (i.e., healthcare provider, faith leader, news outlet, social media, and federal government) were assessed with a 3-point Likert scale using response options of “Not at all,” “A little,” and “A great deal,” with higher mean scores indicating greater trust in that particular information source. Two items also assessed participants’ levels of trust in the U.S. Food and Drug Administration (FDA) and the federal government to ensure COVID-19 vaccine safety generally and specifically for child COVID-19 vaccines. Responses for those two items were assessed using a 3-point Likert scale of “Not at all,” “A little,” and “A great deal,” with higher mean scores indicating greater levels of trust.

### Intervention Exposure and Reactions to Brigada Digital Content.

To assess participants’ exposure to content, they were asked about the source from which they typically received Brigada Digital social media content (i.e., a social media network contact, a social media account for a group you belong to, a social media account for a community health center, someone you don’t know), the frequency with which they received posts (i.e., more than once per day, once per day, a few times per week, once per week, less than once per week), and their typical actions upon receiving posts (i.e., read the post, like/react to the post, comment on the post, share the post, go to a link in the post, follow the post’s advice, attend an advertised event, don’t read the post).

Additionally, to assess reactions to the content, respondents were provided with four statements, including “*Brigada Digital* posts are informative,” “I trust the information that I receive from *Brigada Digital*,” “Posts address my concerns about the COVID-19 vaccine,” and “The way the information was presented in posts kept my interest.” Participants were then asked to indicate the degree to which they agreed/disagreed with these statements using a 5-point Likert scale ranging from “Completely Agree” to “Completely Disagree,” with higher mean scores suggesting greater agreement with the statement.

### Participant Recruitment and Sample.

To assess changes in COVID-19 vaccine–related outcomes of *Brigada Digital* audience members, we administered the CEAL Common Survey 2.0 in Spanish with participants in two waves: May 2022 (n = 480) and April 2023 (n = 348). Eligible participants were Latino adults ages 18 or older who resided in DC or Maryland and who spoke Spanish. Participants were recruited from among parishioners of 3 Maryland-based church partners and from among Maryland- and DC-based social media network members of 10 *Brigada Digital* CHWs. A convenience sample of participants were recruited by sharing a digital flier and making announcements to church congregations, and by *Brigada Digital* CHWs sharing the digital flier in posts to their social media networks. Participants contacted the study team to complete a survey by using a phone number included in the flier.

Only study participants indicating in the survey that they had ever seen a *Brigada Digital* post on a social media platform were included in the present analysis (n = 192 in wave 1; n = 123 in wave 2). Among those exposed to *Brigada Digital* content, 60 individuals in wave 1 and 24 individuals in wave 2 were recruited from among partnering church parishioners and 129 individuals in wave 1 and 98 individuals in wave 2 were recruited from social media network contacts.

### Data Collection.

Following informed consent, surveys were administered in Spanish using an interview format by trained, Latino data collectors in-person with individuals recruited from churches and by phone with individuals recruited from social media networks. Participant responses were entered directly into REDcap by data collectors using a tablet or laptop computer. The survey took approximately 35 minutes to complete, and participants received a $25 gift card incentive. All instruments and protocols were approved by the GW Institutional Review Board.

### Data Analysis.

Study outcomes were assessed among participants who self-reported having been exposed to *Brigada Digital* social media content at waves 1 and 2. To determine the comparability of wave 1 and 2 sample characteristics, we conducted descriptive analyses for socio-demographic, language competency, and health status variables. Chi-squares tests were used for categorical variables and t-tests were used for continuous variables. Means and standard deviations or frequencies and percentages were reported, respectively. In all analyses, the primary independent variable was the wave at which the survey was administered. Subsequent analyses discerned variations in dependent variables as a function of data collection wave.

To assess the primary outcomes of adult vaccine and booster dose uptake, booster intentions, and child vaccine uptake and parent intentions, odds ratios were estimated using logistic regression, while controlling for age, sex, income, language competency, and health insurance status. We controlled for age given its direct correlation with COVID-19 risk levels and the potential influence of age-based vaccine recommendations outcomes for vaccine uptake. Beyond these specific COVID-related reasons, age is generally an important factor to adjust for in health research due to its multifaceted implications on health behavior and outcomes. We also controlled for sex given that women tend to be higher users of healthcare services, including preventative care measures such as vaccination. Additionally, we controlled for income, language competency, and health insurance status since waves 1 and 2 exhibited statistically significant differences for these variables, all of which can influence healthcare access and behaviors.

The survey instrument included items that originally assessed COVID-19 vaccine/booster uptake and intentions concurrently in the same item for adults and children. Therefore, for analytical clarity, these original items were dissected into discrete dummy variables that were generated using response options corresponding to each distinct outcome. This approach permitted a precise portrayal of each distinct outcome; for example, future vaccination intentions could be assessed only among individuals who had yet to be vaccinated with the primary series.

For the secondary outcomes of COVID-19 vaccination subjective norms, difficulty accessing COVID-19 information, trust in COVID-19 information sources and the government, and reactions to *Brigada Digital* social media content, since all variables were assessed using Likert-type response formats, they were treated as continuous variables. Paired t-tests were executed for each variable to discern any differences in these outcomes between waves 1 and 2. For these paired t-tests, a difference was deemed statistically significant if the means differed at a significance threshold of *P* < .05. Means and standard deviations are reported for these variables.

To determine whether there were differences in responses between waves 1 and 2 for participant reasons for not vaccinating children and self-reported exposure to *Brigada Digital* social media content, chi-square tests were performed, with the level of significance demarcated at *P* < .05. Frequencies and percentages are reported for these variables. All analysis was conducted using STATA 17.

## Results

Descriptive statistics for respondents exposed to *Brigada Digital* social media content are presented in Table 1. Most study participants were women, representing 60.9% and 61.8% of individuals at waves 1 and 2, respectively. The mean age of respondents in waves 1 and 2 were similar, at 43.2 years and 42.5 years, respectively.

Approximately 61% of respondents in wave 1 and 70% in wave 2 indicated that they were born outside the U.S., most commonly of Central American origin. There were no statistically significant differences between waves 1 and 2 with respect to country of origin, with El Salvador (*P* = .35), Honduras (*P* = .66) and Guatemala (*P* = .19) being the most common. A majority of respondents indicated that they spoke a language other than English at home (96.3% in wave 1 and 99.2% in wave 2), and moderate levels of English language proficiency were reported at both waves, with participants in wave 2 reporting slightly higher proficiency (M = 2.70, SD = 1.52) than participants in wave 1 (M = 2.25, SD = 1.43) (*P* = .03).

A considerable proportion of participants, 42.1% at wave 1 and 45.5% at wave 2, indicated that they had completed high school, a GED, or had at least some college. Just over three-quarters of respondents indicated that they were employed, and while just over half of wave 1 respondents (54.5%) indicated that they earned an annual household income between $15,000-$34,999, nearly half of respondents (49.6%) declined to answer the question about income at wave 2. Approximately one-third of respondents in waves 1 and 2 reported that they had been diagnosed with a chronic health condition (27.2% and 30.9%, respectively), and while a substantial proportion of individuals reported having health insurance at wave 1 (83.1%), a lower proportion were insured at wave 2 (72.1%) (*P* = .01).

Results for self-reported media consumption are presented in Table 2. A majority of respondents indicated that they spent considerable time navigating the internet, using social media, and viewing videos online, with the most commonly reported amount of time spent doing each of these three activities being 1–3 hours per day.

Almost one-third of participants reported spending three or more hours per day using social media, or 34.9% in wave 1 and 30.1% in wave 2. Similarly, 43.1% of respondents reported viewing videos online three or more hours per day in wave 2, a slight decrease from 48.2% in wave 1.

When asked about social media platforms used, approximately 90% of participants reported using WhatsApp in both waves 1 and 2 (see Table 3).

When asked how much of their COVID-19 information was obtained from specific sources and channels, the most common at both time points included federal or state/local government agencies, followed by social media, friends/family in the U.S., a healthcare provider, and local television/cable news (see Table 4).

### Primary Outcomes.

A significant increase was observed from wave 1 to wave 2 in respondents indicating that they (OR = 6.48, 95% CI [2.73, 15.33]) or their children (OR = 6.00, 95% CI [2.15, 16.64]) had received the primary COVID-19 vaccine series. Respondents from wave 2 had 6.48 times the odds of having received the COVID-19 vaccine themselves, and six times the odds of having had the vaccine administered to their child(ren) (see Table 5).

With regard to the proportion of respondents reporting having received the COVID-19 primary vaccine series, at wave 2, 92.7% of respondents reported being vaccinated, compared to 61.9% in wave 1. A similar upward trend was observed for child vaccination, with 73.6% of respondents indicating they had vaccinated their child(ren) against COVID-19 in wave 2, compared to 32.4% in wave 1. Both of these outcomes showed statistically significant differences from wave 1 to 2 (*P* < .001). For booster dose uptake, 78.8% of adult respondents in wave 2 had received a booster dose, compared to 64.0% in wave 1. While there were increases in adult booster dose uptake and intentions to receive a booster dose from wave 1 to wave 2, these results were not statistically significant. Respondents who had never received a COVID-19 booster dose (n = 60 across both waves) were asked to provide reasons for their decision. The most common reasons given for having not yet received the booster dose included not wanting to have secondary side effects (51.7%) and a belief that a booster dose was unnecessary (30.0%).

Among respondents who had not yet vaccinated their child(ren) against COVID-19, there was a significant increase from wave 1 to wave 2 in respondent intentions to vaccinate their child(ren) under age 18 (OR = 4.81, 95% CI [1.66, 13.93]). Respondents who had not yet vaccinated their child(ren) against COVID-19 were asked to provide reasons for this decision (n = 64 across both waves). The most common reasons given by parents/guardians are presented in Table 6.

The most cited reason parents endorsed for not vaccinating their child(ren) against COVID-19 was concern about side effects (14.9%). However, this was significantly less likely to be listed as a concern among wave 2 (9.8%) compared to wave 1 (18.2%) respondents (*P* = .04). Other common reasons included mistrust in the development process for the vaccine (7.3%), concern about vaccine efficacy for children (6.3%), and a belief that the vaccine is not needed because children are at low risk of becoming seriously ill from COVID-19 (6.3%). However, there were statistically significant decreases from wave 1 to wave 2 in these concerns about the COVID-19 vaccine development process and safety, as well as the belief that children are at low risk for severe illness as reasons for not vaccinating.

### Secondary Outcomes.

Perceptions of COVID-19 vaccine uptake as being normative increased overall during the study period (see Table 7).

There were statistically significant increases in participant perceptions that people close to them thought they should get the vaccine from wave 1 (M = 2.67, SD = 1.20) to wave 2 (M = 3.00, SD = 1.02), with P < .05. Participants also perceived that more of their family members had received the COVID-19 vaccine in wave 2 than in wave 1, with a mean score of 3.17 (SD = 0.88) in wave 2, a statistically significant increase compared to a mean score of 2.72 (SD = 0.65) in wave 1, with *P* < .001.

When asked about perceived difficulty finding COVID-19 information in their preferred language, respondents reported a slight decrease in perceived difficulty from wave 1 (M = 1.36, SD = 0.54) to wave 2 (M = 1.35, SD = 0.66), but results were not significant (see Table 8).

Additionally, respondents reported the most difficulty in judging the reliability of COVID-19 information in the media; however, a statistically significant decrease in perceived difficulty was observed from wave 1 (M = 2.97, SD = 0.92) to wave 2 (M = 2.50, SD = 1.17) (*P* < .001). Despite these improvements, participant responses indicated a slight increase in perceived difficulty finding needed COVID-19 information from wave 1 (M = 1.42, SD = 0.61) to wave 2 (M = 1.54, SD = 0.86), though this increase was not statistically significant.

With regards to trust in different COVID-19 information sources, respondents across both waves reported having the greatest trust in healthcare providers compared to other sources, with a mean score of 2.47 (SD = 0.53) in wave 1 and 2.55 (SD = 0.53) in wave 2 (see Table 9).

Respondents were also asked about their level of trust in the federal government to ensure COVID-19 vaccine safety for adults and children. There was a statistically significant increase for trust in the FDA to ensure COVID-19 vaccine safety from wave 1(M = 2.02, SD = 0.87) to wave 2 (M = 2.37, SD = 0.74) (*P* < .001), and a significant increase for trust in the federal government to ensure child COVID-19 vaccine safety from wave 1 (M = 1.87, SD = 0.88) to wave 2 (M = 2.30, SD = 0.76) (*P* < .001).

Respondents also reported having high levels of trust in the Centers for Disease Control and Prevention (CDC). The least trusted sources of information included contacts on social media, work, school, or other acquaintances, and radio, tv, internet, or print news sources. However, trust in all listed COVID-19 information sources increased significantly from wave 1 to wave 2, with the exception of healthcare providers, for which the mean score did not differ significantly between waves.

### Exposure and Reactions to Brigada Digital Content.

The frequency of exposure to *Brigada Digital* content, the source of this content, audience self-reported reactions, and actions they took in response to the content were assessed in waves 1 and 2 (see Table 10).

The most likely channel through which participants were exposed to content was a personal social media contact (38.4%), followed by messages disseminated via church (23.8%) and community health center (17.8%) partner social media accounts. In terms of self-reported frequency of exposure to *Brigada Digital* content, about 24.8% of respondents indicated seeing this content a few times per week, while 27% indicated exposure once or more per day. Overwhelmingly, the most common action taken in response to seeing content was to read the post (96.5%), with the next most common response being to share the content to which they were exposed (22.5%). Almost one-tenth of respondents reported moderate levels of engagement with the content, such as commenting on (9.8%) or liking/reacting (8.2%) to a post.

Participant reactions to *Brigada Digital* content were assessed among wave 2 participants only. Overall, participants reported positive reactions to the content (see Table 11).

## Discussion

Results demonstrated that study participants who were exposed to *Brigada Digital* content were avid consumers of online content, with a considerable proportion of individuals reporting spending hours per day navigating the internet, watching videos online, and using social media. Other studies have shown similar patterns, including high Facebook and YouTube use among Latinos nationally, with 72% reporting using Facebook and 85% reporting using YouTube in 2021 (40), compared to 76.4% of study participants using Facebook and 95.1% using YouTube in 2023. Use of the WhatsApp and TikTok platforms have also shown tremendous growth in recent years, as demonstrated by 90.2% and 45.5% of respondents, respectively, reporting using these platforms in 2023, compared to 46% and 31% of Latinos reporting using these platforms nationally in 2021. Respondents tended to be predominantly women between the ages of 26–55, which mirrors the audience that follows *Brigada Digital* social media accounts (39). Study participants also tended to have slightly higher educational levels in 2023 than the national average, with 83.7% having completed high school/GED or higher, compared to 75.4% of U.S. Latino adults in 2021 (41). With regards to health insurance status, more study respondents (27.9%) reported being uninsured in 2023 compared to than Latinos at the national level in 2021 (17.7%).

When asked about levels of trust in different sources of COVID-19 information, trust increased overall from wave 1 to wave 2, which may potentially be reflective of respondents’ increasing familiarity with COVID-19 as a risk and healthcare providers’ and government entities’ ability to disseminate information to communities as more was learned about the virus and vaccines. Interestingly, study results showed that while respondents trusted healthcare providers, the CDC, and federal/state governments the most as sources of COVID-19 information, a finding demonstrated by other studies (42–44), the sources from which they reported obtaining a greater proportion of their COVID-19 information included social media, television news outlets, and federal/state governments. Additionally, at both time points, respondents said they received a greater proportion of their information from social media than from their healthcare providers, and in wave 2, social media outpaced news outlets and state/local governments as a source of COVID-19 information. Similarly, a 2021 study reported that Spanish-speaking Latinos in Washington state obtained more COVID-19 information from television than healthcare providers, followed by social media and community-based organizations (45). Another 2021 study also found that among Pittsburgh-area Black and Latino respondents, the most frequently used COVID-19 information sources were the local TV news (66%) or friends and family (64%), followed by, among other sources, the national news (59%), the CDC (49%), local doctors (48%), and the county health department (45%) (46). Furthermore, while significant decreases were observed in participants’ perceived difficulty in judging the reliability of COVID-19 information in the media, study results signaled that judging information reliability continued to be a challenge for participants in wave 2. These findings provide further evidence in support of approaches like *Brigada Digital* that aim to increase access to reliable COVID-19 information in Spanish and to build community capacity to distinguish between reliable and unreliable health information in digital environments. Patterns of media consumption and COVID-19 information seeking among participants in this study also highlight the relevance of digital strategies for health promotion using trusted messengers, in particular healthcare providers and CHWs.

With regards to COVID-19 vaccine outcomes, study results showed that, among respondents who were exposed to *Brigada Digital* content, there were significant differences between the responses of wave 1 and wave 2 participants across primary COVID-19 vaccine-related outcomes. Specifically, respondents in wave 2 were significantly more likely to have received the initial COVID-19 vaccine series (92.7%) compared to respondents in wave 1 (61.9%). This represents a level of vaccine uptake that is substantially higher than Latinos nationwide - only 57% of U.S. Latino adults had completed a primary COVID-19 vaccine series as of May 2023, close to the time of wave 2 survey administration (13). Additionally, 73.6% of study respondents indicated that they had vaccinated their child(ren) under age 18 against COVID-19, yet only 28.8% of U.S. Latino children ages 5–11 years, 57.8% of those ages 12–15 years, and 70.4% of those ages 16–17 years had completed the vaccine series by August 2023 (15).

Additionally, there was only modestly higher booster dose uptake at wave 2 compared to wave 1, and these differences were not significant. This lack of significance is likely explained by a relatively high rate of booster dose uptake at both time points, or 64.0% at wave 1 and 78.8% at wave 2. Again, this level of uptake far exceeds booster dose uptake by Latino adults nationwide, which was 8.5% as of May 2023 (14). These higher levels of vaccine series and booster uptake among study respondents compared to Latinos nationally likely signal potential disparities across different Latino communities and U.S. regions that merit further investigation. This result may also reflect a high level of prioritization of health and endorsement of COVID-19 vaccination among audiences who follow the *Brigada Digital de Salud* on social media, or who are connected with community health workers or partner organizations in social media networks.

Respondents who had received *Brigada Digital* content reported being reached predominantly through personal contacts on social media, their church’s social media account, or the account of a community health center. These channels reflect our primary outreach and engagement strategies through individuals and institutions that are known and trusted, including CHWs and community-based organizations. Approximately 41% of respondents indicated that they were exposed to *Brigada Digital* content a few times per week to once per day, which is consistent with the frequency with which we disseminated content to Brigada *Digital’s* main accounts. For individuals who were not following *Brigada Digital* pages directly, this frequency of exposure likely varied depending on the frequency with which each CHW shared and reposted content to their social media networks. While the majority of respondents (96.5%) said that they read *Brigada Digital* posts when received, fewer respondents shared the content with their networks (22.5%), commented (9.8%), or liked/reacted (8.6%). Future efforts should identify strategies to further augment audience engagement with similar content. Generally speaking, respondents who were exposed to this content reacted positively, finding the content to be informative, trustworthy, addressing COVID-19 vaccine concerns, and delivered in an interesting way. Given the overall higher levels of mistrust of COVID-19 information on social media, this reported trust in *Brigada Digital* content can possibly be explained by the information sources being known and trusted individuals or community organizations, and the content being culturally- and linguistically-appropriate.

### Limitations.

There were several limitations that should be considered when interpreting study results. The study employed a one-group repeated cross-sectional design. Given that the survey was administered to different individuals in waves 1 and 2, it is difficult to make definitive statements about changes in outcomes over time. Study participants included a modest sample size of Latino adults from the Washington, DC metropolitan area who were predominantly foreign-born and originating from Central American countries, suggesting that results may not be generalizable to all U.S. Latino subgroups. Given that a non-probability-based sampling strategy was used, this introduced the possibility of selection bias, in that individuals who were willing to engage with digital media content as a conduit for health messaging or participate in a health-related survey may share a tendency towards greater interest in and commitment to their health in general. Further, exposure to *Brigada Digital* material was self-reported, making it difficult to precisely quantify levels of exposure. Finally, given that information about COVID-19 was available prior to the study and became increasingly available as the pandemic progressed, it is difficult to differentiate between changes attributable to the intervention and those which may have resulted from exposure to other sources of information. Taken together, these limitations invite further study in an effort to more firmly establish links between digital health messaging and COVID-19 vaccine-related outcomes. It is important to note that outcomes described here are the result of a pilot study, and therefore present a number of opportunities for further research. Study results suggest the importance of social media as a tool for the dissemination of reliable health information, which may be particularly useful in reaching communities that commonly experience barriers to accessing health-related information and services. Results also underscore the importance of identifying and utilizing trusted sources for the delivery of information, and the ways in which the delivery of accurate health information is linked with changes in health behaviors, intentions and perceived behavioral norms.

## Conclusions

The *Brigada Digital de Salud* was established to reduce Spanish language COVID-19 information access barriers and combat misinformation on social media that fuel vaccine hesitancy among Latino audiences. Importantly, this effort leveraged community-based strategies and reputable, familiar sources of information to build trust and promote vaccine uptake. This study offers important insights into Latino audience segments that can be reached using similar digital strategies, as well as audience reactions to culturally- and language-appropriate social media content promoting COVID-19 risk mitigation. While further research is needed to test the digital community-based approach, study results show promise in terms of vaccine-related behaviors, intentions, and perceptions. Future research that seeks to employ a similar approach should aim to build community capacity to conduct digital community-based outreach and health promotion, and build community capacity to navigate the complex health information environment to locate reliable health information through improved digital health literacy.

## Figures and Tables

**Table 1:** Participant Sociodemographics and Characteristics

Variables	Wave 1 (*n* = 192)		Wave 2 (*n* = 123)		*P* value
	n	%	n	%	
**Age Groups**					.26
18–25	35	18.2	16	13.0	
26–35	32	16.7	32	26.0	
36–45	33	17.2	18	14.6	
46–55	46	23.9	36	29.3	
56–63	21	11.0	8	6.50	
64+	20	10.4	11	8.9	
Not reported	5	2.60	2	1.63	
**Sex**					.88
Male	75	39.1	47	38.2	
Female	117	60.9	76	61.8	
**Birthplace**					.13
US	75	39.1	38	30.9	
Non-US	116	60.4	85	69.1	
Missing	1	0.52	0	0.00	
**Level of Education**					.64
Less than HS, some HS	36	18.9	18	14.6	
HS grad, GED, some college	80	41.9	55	44.7	
Associate, bachelor, postgraduate	74	38.7	48	39.0	
Missing	1	0.52	2	1.63	
**Employment status**					.25
Employed	151	78.7	95	77.2	
Unemployed	16	8.3	6	4.9	
Other (disabled, student, retired)	25	13.0	22	17.9	
**Household Income**					< .001***
<$15,000–$34,999	104	54.2	19	15.5	
$35,000–$74,999	48	25.0	15	12.2	
$75,000–$100,000>	33	17.2	28	22.8	
Declined to answer	7	3.7	61	49.6	
**Have health insurance** *	159	83.1	88	72.1	.01*

* = *P* value < .05

** = *P* value < .01

*** = *P* value < .001

**Table 2:** Daily Media Consumption

Time spent on an average day...	Wave 1 (*n* = 192)		Wave 2 (*n* = 123)		*P* value
	n	%	n	%	
**Navigating the Internet**					.001***
None	0	0.0	2	1.6	
<1 hour	23	12.0	16	13.0	
1–3 hours	65	33.9	58	47.2	
3–6 hours	47	24.5	34	27.6	
6 + hours	55	28.6	13	10.6	
Not reported	2	1.0	0	0.0	
**Viewing Videos Online**					.007**
None	11	5.7	0	0.0	
<1 hour	33	17.2	17	13.8	
1–3 hours	55	28.6	53	43.1	
3–6 hours	53	27.6	36	29.3	
6 + hours	39	20.3	17	13.8	
Not reported	1	0.5	0	0.0	
**Using Social Media**					.26
None	8	4.2	2	1.6	
<1 hour	32	16.7	15	12.2	
1–3 hours	85	44.3	69	56.1	
3–6 hours	50	26.0	28	22.8	
6 + hours	17	8.9	9	7.3	
Not reported	0	0.0	0	0.0	

* = *P* value < .05

** = *P* value < .01

*** = *P* value < .001

**Table 3:** Social Media Platforms Used

Platforms					
	Wave 1 (*n* = 192)		Wave 2 (*n* = 123)		*P* value
	n	%	n	%	
Facebook	163	84.9	94	76.4	.06
Instagram	111	57.8	63	51.2	.25
Twitter	43	22.4	17	13.8	.06
WhatsApp	174	90.6	111	90.2	.91
YouTube	171	89.1	117	95.1	.06
Snapchat	43	22.4	22	17.9	.33
TikTok	69	35.9	56	45.5	.09

* = *P* value < .05

** = *P* value < .01

*** = *P* value < .001

**Table 4:** Proportion of COVID-19 Information Obtained from Various Sources and Channels

COVID-19 Information Sources/Channels	Wave 1 (*n* = 192)		Wave 2 (*n* = 123)		*P* value
	M^a^	SD	M^a^	SD	
Healthcare Provider	1.84	0.78	1.95	0.84	.27
Print News (e.g. Washington Post)	1.17	0.38	1.16	0.43	.93
Local TV News (e.g. NBC, ABC)	1.71	0.66	2.05	0.72	< .001***
Cable TV (e.g. CNN, FOX, MSNBC)	1.66	0.65	2.07	0.72	< .001***
Local Radio Station	1.43	0.65	1.40	0.64	.62
Online News Sites	1.53	0.66	1.94	0.90	< .001***
Social Media	1.96	0.61	2.29	0.72	< .001***
Friends/Family in the US	1.92	0.58	2.06	0.58	.05
Friends/Family outside the US	1.49	0.59	1.73	0.70	.001**
Church Contacts	1.70	0.63	1.49	0.58	.003**
State or Local Government	2.09	0.69	2.16	0.69	.39
Federal Gov’t Agencies (e.g. CDC, NIH)	2.22	0.69	2.45	0.74	.005**
President of the United States	1.60	0.67	1.59	0.61	.82

* = *P* value < .05

** = *P* value < .01

*** = *P* value < .001

a Proportion of COVID-19 information is expressed with a mean score (range 1–4), with a higher value indicating greater proportion of COVID-19 information obtained from the source.

**Table 5:** Adjusted Odds Ratio of COVID-19 Vaccine/Booster Dose Uptake and Intention

Variable	Wave 2		
	Adjusted OR^a^	95% CI	*P* value
Received vaccine	6.48	2.73, 15.33	< .001***
Received booster dose	1.78	0.86, 3.64	.12
Intention to receive booster dose^b^	1.25	0.59, 2.61	.56
Child(ren) received vaccine	6.00	2.16, 16.65	< .001***
Intention to vaccinate child(ren)	4.81	1.66, 13.93	.004**

* = *P* value < .05

** = *P* value < .01

*** = *P* value < .001

a Odds ratios adjusted for income, sex, age, language, and health insurance status.

b Responses from participants who have not yet received a booster dose.

**Table 6:** Reasons for Not Vaccinating Child(ren) Against COVID-19

Reasons	Wave 1 (*n* = 50)		Wave 2 (*n* = 14)		Combined (*n* = 64)		*P* value
	n	%	n	%	n	%	
My children are in a low-risk group for severe illness from COVID-19	18	9.4	2	1.6	20	6.3	.006**
I don’t trust the process by which the COVID-19 vaccine was developed	20	10.4	3	2.4	23	7.3	.008**
I’m worried about side effects of the vaccine in children	35	18.2	12	9.8	47	14.9	.04*
I don’t think the vaccines work well for children	16	8.3	4	3.3	20	6.3	.07

* = *P* value < .05

** = *P* value < .01

*** = *P* value < .001

**Table 7:** COVID-19 Vaccination Subjective Norms

Subjective Norms	Wave 1 (*n* = 192)		Wave 2 (*n* = 123)		*P* value
	M^a^	SD	M^a^	SD	
People close to me think I should get the COVID-19 vaccine	2.67	1.20	3.00	1.02	.01*
My friends have received the COVID-19 vaccine	2.79	1.05	2.92	0.75	.21
My family members have received the COVID-19 vaccine	2.72	1.17	3.17	0.88	.001***
My community members have received the COVID-19 vaccine	2.88	0.65	2.92	0.48	.45

* = *P* value < .05

** = *P* value < .01

*** = *P* value < .001

a Perceived norms are expressed with mean scores (range 1–4), with higher scores indicating greater perception in the indicated norm.

**Table 8:** Perceived Difficulty Accessing COVID-19 Information

Perceived Difficulty Statements	Wave 1 (*n* = 192)		Wave 2 (*n* = 123)		*P* value
	M^a^	SD	M^a^	SD	
Difficulty finding needed COVID-19 information	1.42	0.61	1.54	0.86	.12
Difficulty finding COVID-19 information in preferred language	1.36	0.54	1.35	0.66	.88
Difficulty judging whether COVID-19 information in the media was reliable or not	2.97	0.92	2.50	1.17	< .001***

* = *P* value < .05

** = *P* value < .01

*** = *P* value < .001

a Perceived difficulty is expressed with a mean score (range 1–4), with higher values indicating greater difficulty.

**Table 9:** Trust in COVID-19 Information Sources and Agencies Overseeing Vaccine Safety

Level of Trust in...	Wave 1 (*n* = 192)		Wave 2 (*n* = 123)		*P* value
	M	SD	M	SD	
**Information Sources**					
Healthcare Provider	2.47	0.53	2.55	0.53	.21
Religious/Spiritual Leader (e.g. pastor)	2.04	0.80	2.31	0.61	< .001***
Work, School, or other Acquaintances	1.52	0.58	2.00	0.53	< .001***
News-Radio, TV, Internet, or Print	1.62	0.63	2.00	0.61	< .001***
Social Media Contacts	1.64	0.59	1.94	0.60	< .001***
Federal Government	1.96	0.81	2.22	0.65	.003**
State or Local Government	1.97	0.82	2.23	0.65	.003**
Centers for Disease Control and Prevention	2.14	0.81	2.42	0.64	.002**
A Community Organization	1.83	0.63	2.09	0.48	.001***
**Agencies Overseeing Vaccine Safety**					
Trust federal gov’t to ensure COVID-19 vaccine safety	2.02	0.87	2.37	0.74	.001***
Trust FDA to ensure COVID-19 vaccine safety for children	1.87	0.88	2.30	0.76	.001***

* = *P* value < .05

** = *P* value < .01

*** = *P* value < .001

a Trust in COVID-19 information source is expressed with a mean score (range 1–3), with a higher value indicating greater trust in the source.

**Table 10:** Brigada Digital Content Exposure Source, Frequency and Actions Taken

	Waves 1 and 2 (*n* = 315)	
	n	%
**Source of Exposure to *Brigada Digital***		
Personal social media contact	121	38.4
Church social media account	75	23.8
Social media account for group you belong to	43	13.7
Social media account for community health center	56	17.8
From someone you did not know	20	6.4
**Frequency of Posts Seen**		
More than Once per Day	34	10.8
Once per Day	51	16.2
A few times per week	78	24.8
Less than once per week	67	21.3
Don’t Know	85	27.0
**Actions Taken After Seeing Post**		
Read Post	304	96.5
Liked/Reacted	27	8.6
Commented on	31	9.8
Shared/Retweeted	71	22.5
Visit the Link	5	1.6
Follow Advice in Post	9	2.9
Attend an Event	4	1.3
Did Not Read Post	1	0.3
Deleted Post	0	0.0

**Table 11:** Reactions to Brigada Digital Content

Statements about *Brigada Digital* Content	Wave 2 (*n* = 123)	
	M^a^	SD
*Brigada Digital* posts were informative.	3.95	0.73
I trust the information I received from *Brigada Digital*.	3.72	1.02
*Brigada Digital* posts addressed my COVID-19 vaccine concerns.	3.91	0.70
The way *Brigada Digital* delivered information kept me interested.	3.74	0.99

a Reactions are expressed with a mean score (range 1–5), with higher values indicating greater agreement with statements about *Brigada Digital* content.

## Abbreviations

CHW: Community Health Worker
CDC: Centers for Disease Control and Prevention
NIH: National Institutes of Health
CEAL: Community Engagement Alliance Against COVID-19 Disparities
DMV: Washington DC Metropolitan Area
FDA: Food and Drug Administration
GED: General Education Development HS: High School
